# Editorial: Materials for Electroanalysis Based on Advanced Frameworks

**DOI:** 10.3389/fchem.2021.638338

**Published:** 2021-02-15

**Authors:** Baiqing Yuan, Dong Liu, Huajie Yin, Daojun Zhang

**Affiliations:** ^1^ School of Chemistry and Materials Science, Ludong University, Yantai, China; ^2^ Key Laboratory of Modern Agricultural Equipment and Technology, Ministry of Education, School of Agricultural Equipment Engineering, Jiangsu University, Zhenjiang, China; ^3^ Centre for Clean Environment and Energy, Griffith University, Southport, QLD, Australia; ^4^ Henan Province Key Laboratory of New Optoelectronic Functional Materials, College of Chemistry and Chemical Engineering, Anyang Normal University, Anyang, China

**Keywords:** covalent organic frameworks (COFs), metal-organic frameworks (MOFs), electrochemical sensors/sensing, electrical conductivity, photoelectric conversion, near-infrared phosphorescence, nanoscale

**Editorial on the Research Topic**

**Materials for Electroanalysis Based on Advanced Frameworks**

Covalent organic frameworks (COFs) and metal-organic frameworks (MOFs) are two emerging classes of extended porous structures, which seek to develop the reticular chemistry beyond molecules and open up new horizons for compositions, structures, properties, and applications (Yaghi, 2019; Lyu et al., 2020). Like MOFs that extend inorganic metal complexes into 2D and 3D frameworks, COFs extend organic chemistry from molecules and polymers into 2D and 3D organic structures (Diercks and Yaghi, 2017). MOFs/COFs are built with the aim of extending porous frameworks through strong bonds (coordinate/covalent interactions) between molecular building blocks (metal-containing unit-organic linker/organic-organic monomer) based on topological guides. The advantages of these approaches include controllable synthesis, pre-designable structures, and manageable functionality (Geng et al., 2020). In addition to possessing high surface area and tunable pores, both MOFs and COFs display a lot of intriguing properties including layered crystalline structures through π-π stacking and high stability which is only exhibited in graphene (Fritz and Coskun, 2020) owing to the presence of strong covalent bonds. However, metal-free COFs far from meet the growing demands of numerous fields where the role of metal in the framework structure is emphasized. This includes applications such as gas adsorption and separation, heterogeneous catalysis, electronics, electrocatalysis, and electrochemical energy storage. An effective way to address these challenges is to introduce targeted metal ions into COFs frameworks to form metal-covalent organic frameworks (MCOFs) (Dong et al., 2020). Compared with metal-free COFs, MCOFs not only have superior electrocatalytic activity but also display higher intrinsic conduction due to the involvement of the metal component. Developing distinctive synthesis methods/strategies to achieve novel MOFs and COFs holds much promise toward promoting their application. For instance, flexible and free-standing pure COFs membranes were prepared by liquid-liquid interfacial polymerization at room temperature and atmospheric pressure, which solves a major problem since COFs are generally insoluble and unprocessable powders (Liu et al., 2020). A vast number of organic monomers have been reported to date with infinite possibilities of functionalization in their resulting structures. This leads to “digital reticular chemistry” based on laboratory robotics and artificial intelligence (AI), which could achieve high-throughput experiments involving synthesis and characterization. This approach is poised to make the discoveries in MOFs and COFs more significant and easily achievable (Lyu et al., 2020).

Since the first report in 1962 for the detection of glucose using glucose oxidase, electrochemical sensing has been well accepted as a powerful tool in a variety of fields where high sensitivity, simple operation, rapid response, and low cost are necessary. Electrochemical sensing is especially suited for miniaturization, and therefore offers multiple construction merits for manufacturing flexible, disposable, and cheap devices (Amiri et al., 2018). The introduction of novel elements into MOFs and COFs brings enhanced scope for electrochemical sensing, which promise to be a major boost toward their synthesis. For example, MOFs and COFs possess highly ordered porous structures, adjustable holes, and manageable functionality, thereby providing an efficient and powerful platform to anchor electroactive molecules, biomolecules, and nanoparticles. Yet, there are still many key issues regarding MOFs and COFs that need to be urgently resolved before they can be successfully applied in electrochemical sensing. In this context, Yuan et al. presented a critical review on the recent advances regarding COFs and their applications in electrochemical sensing, with a focus on the mechanism and methods/strategies for improving electrical conductivity, the immobilization of COFs on different substrates, device miniaturization, and application in electrochemical sensors. On this Research Topic, we present eight original research articles and one review article to showcase the recent advances in MOFs and COFs explored for electrochemical sensing.

A key problem regarding the application of MOFs and COFs in electrochemical sensing is the inherent low conductivity of bulk MOFs and COFs. It essentially implies that porosity and high surface area do not coexist with excellent electrical conductivity which is indispensable for fast electron transfer in electro-analysis. However, most of these framework materials are typically poor electrical conductors owing to the lack of transport channels for the charge carriers. Neither hopping transport nor band transport pathways are feasible. This result mainly originates from the intrinsically insulating character of the organic ligands, as well as the poor energetic and orbital overlap between the ligands and metal-oxo nodes (e.g., π−d interactions) (Sun et al., 2016; Pratik et al., 2020). The types of linkages (e.g., imine, imide, and boroxine) that are formed while building conventional COFs decides that the charge transport in COFs follows an interlayer hopping mechanism rather than an in-plane mechanism, resulting in wide band gaps and low in-plane conduction. In-plane conduction is improved by conducting polydisperse 1D graphene nanoribbons (GNRs)-based COFs via interfacial polymerization (Veber et al., 2020). Luo et al. report a 2D conductive nano-MOF (NiCu-CAT) composed of a 2, 3, 6, 7, 10, 11-hexahydroxytriphenylene ligand that shows high electrocatalytic activity toward the oxidation of paracetamol. The full in-plane charge delocalization is achieved by π-π stacking, which contributes to the excellent electrical conductivity. A conductive Ni_3_ (2, 3, 6, 7, 10, 11-hexaiminotriphenylene)_2_ MOF is explored by Wu et al. as an electrochemical sensing platform with long-term stability, for the detection of glucose. Another efficient strategy to enhance electrical conductivity is to mix conductive materials with MOFs or COFs to form conductive composites. For instance, Song et al. fabricated conductive COFs composites based on nitrogen-contained carbon derived from kenaf stem. The conductive composites are demonstrated to immobilize acetylcholinesterase for the detection of organophosphorus pesticides over a wide linear range, with a low detection limit (0.067 ng/ml). Karimi-Maleh et al. report the development of a highly sensitive electrochemical biosensor for analyzing the mitoxantrone anti-cancer drug by using DNA as a recognition moiety based on ZIF-8/ionic liquid carbon paste electrodes. The presence of ZIF-8 helps in the high loading of ds-DNA, and BMIM serves as the conductive binder for improving the electrical conductivity and sensitivity of the sensor. Combination of porosity and high electrical conductivity can also be accomplished by derivatization of MOFs or COFs. Derived MOFs or COFs can also enhance the structural stability. Pang et al. demonstrate Ni/NiO heterostructure nanobelts derived from Ni-based MOF for use as an electrochemical platform for the sensitive detection of glucose. This derived MOF exhibits an interleaved 3D reticulated structure with high mechanical stability. Besides, ultrafine Ni nanoparticles decorated on the NiO nanobelt enhance the electrical conductivity and active sites.

MOFs-based materials possessing both strong enrichment ability and high electrocatalytic activity are demonstrated by Shirsat et al. The incorporation of Au nanoparticles in CuBTC MOFs is used for the sensitive detection of Pb^2+^. Other contributions are about the synthesis of a redox-active UV-11 MOF and its characterization by cyclic voltammetry, by Cobos-Murcia et al., and near-infrared phosphorescence emission by binuclear Mn(II)-based MOFs for efficient photoelectric conversion, by Zhang et al.

Though limited articles are collected in this topical issue due to the COVID-19 pandemic, we hope that this Research Topic will serve as a guide for the novel design and modification of frameworks explored in the field of electrochemical sensing. We think the following research/challenges highlighted in this issue may be of interest to readers in terms of the application of frameworks or their composites/derivatives in electrochemical sensing: structures with both large porosity and high conductivity, simple immobilization methods/strategies developed for MOFs/COFs on different conductive substrates, hydrogels/aerogels/flexible films to overcome intrinsic fragility and powdered crystalline states of MOFs/COFs, nanoscale MOFs/COFs, encapsulation of biomolecules in MOFs/COFs to enhance thermal, chemical, and mechanical stability, structures possessing enzyme-mimicking activity, stimuli-responsive frameworks, and enantioselective/chiral sensing. Finally, we would like to thank all the authors for their contributions to this collection. We also appreciate the referees and the editorial staff of Frontiers in Chemistry for their efforts toward in the publication of this topical issue.
